# The Improving Role of Basalt Fiber on the Sulfate–Chloride Multiple Induced Degradation of Cast-In-Situ Concrete

**DOI:** 10.3390/ma17184454

**Published:** 2024-09-11

**Authors:** Yiqi Hu, Zhuo Wang, Zhilong Chen, Cheng Wang, Shijun Ding, Zhibao Nie, Tianxin Hou, Gaowen Zhao

**Affiliations:** 1 School of Highway, Chang’an University, Xi’an 710064, China; oscarhyq@163.com (Y.H.); 2021121088@chd.edu.cn (Z.W.); 2022121118@chd.edu.cn (Z.C.); 2022121124@chd.edu.cn (T.H.); 2 Key Laboratory for Special Area Highway Engineering, Ministry of Education, Chang’an University, Xi’an 716400, China; 3 College of Water Conservancy and Architectural Engineering, Northwest A&F University, Yangling 712100, China; wchgghwzy@163.com; 4 China Electric Power Research Institute, Beijing 100085, China; acmilan03@126.com (S.D.); cstudyx@163.com (Z.N.)

**Keywords:** cast-in-situ concrete, degradation mechanism, sulfate attack, magnesium attack, basalt fiber

## Abstract

In salt lake areas, the cast-in-situ concrete structure has been corroded by the combination of sulfate and chloride for a long time. The incorporation of basalt fiber materials into concrete helps to improve the durability of concrete. In this paper, experiments were conducted to study the corrosion deterioration mechanisms of basalt fiber-reinforced cast-in-situ concrete under sulfate, chloride, and combined attack. The appearance, size, mass, flexural, and compressive strength of specimens were investigated during the immersion period to determine the changes in the physical and mechanical properties of specimens. Moreover, the microstructure and mineral changes of specimens during the immersion period were observed by Scanning Electron Microscope (SEM), Energy Dispersive Spectrometer (EDS), X-ray diffraction (XRD), and Thermogravimetric (TG)/ Derivative Thermogravimetric (DTG) analyses. Results show that premixed chloride has a significant detrimental influence on the strength development of cast-in-situ concrete, with concrete powder spalling occurring on the surface of the specimen. Severe corrosion degradation of specimens occurs under the external sulfate and internal chloride combined attack, resulting in lower flexural and compressive strength. The compressive strength and flexural strength of the corroded specimens decreased by 15.4% and 24.8%, respectively, compared with the control group at 28 days. Moreover, premixed basalt fiber has a beneficial influence on cast-in-situ concrete. When the basalt fiber content is 0.5%, the flexural strength of the specimen is increased by 16.2%. The filling and bridging effect of basalt fiber alleviates the negative effects caused by corrosion. In addition, increasing fiber content is beneficial for enhancing its effectiveness when the fiber content is less than 0.5%. This paper provides a valuable reference for the application of basalt fiber-reinforced cast-in-situ concrete under the condition of sulfate–chloride compound corrosion.

## 1. Introduction

Cement-based materials such as concrete are widely used due to their high processability, low cost, and high strength. However, concrete also suffers from drawbacks such as high brittleness, high self-weight, and susceptibility to cracking [1]. After the concrete is poured, its internal capillary pressure increases with water evaporation, leading to shrinkage cracks formation at the early stage [2,3]. During the service period, the expansion of early cracks under the coupled effects of loading and environment would negatively affect the physical and mechanical properties of concrete. Expansion of cracks would also increase the porosity of concrete, which reduces the durability of concrete and leads to a serious reduction in the service life and safety of structures [4,5,6].

In addition, cement-based materials have significant durability drawbacks when serving in environments that are rich in acids or corrosive ions [7]. The soil and groundwater in salt lakes, salt soils, and coastal areas contain high levels of aggressive ions such as sulfate, chloride, and magnesium [8,9,10]. Concrete structures in these areas (e.g., pile foundations, bridge pillars, and submarine tunnels) are susceptible to corrosion by groundwater, soil, and seawater. This may cause deterioration of the structural load-bearing characteristics and shortening of the service life of the structure [11,12,13]. Therefore, improving the corrosion resistance of concrete is an urgent problem. Researchers have been exploring the corrosion deterioration mechanism of concrete and searching for alternative materials for the sustainable construction industry [14,15,16], such as using supplementary cementing materials (SCMs) to partially replace cement to improve concrete corrosion resistance. Common SCMs include fly ash, silicon powder, and mineral powder [17,18,19]. However, due to the uncertainty of the structural service environment and the complexity of the reaction between SCMs and cement, selecting and determining the type and amount of SCMs is very complex [20,21].

In recent years, researchers have shown that materials such as polypropylene or modified polypropylene fiber [22], fly ash, and nano-silica have a positive effect on the structure and parameters of concrete [23]. However, the good engineering properties of the fiber and the anti-cracking and enhanced toughness effect of fibers on concrete have attracted people’s interest [24,25,26]. Using fibers to prepare fiber-reinforced concrete is a feasible method to improve the durability of concrete. Basalt fiber (BF) has been widely used to prepare basalt fiber-reinforced concrete (BFRC) due to its cost-effectiveness, high corrosion prevention, and high-temperature resistance [27,28,29,30,31]. Sohail et al. [32] and Zeng et al. [33] conducted experiments to study the effects of BF on the specimen and found that BF can inhibit crack growth and improve the compressive strength of the specimen. Zhou et al. [34] conducted experiments to study the compressing, tensile, and bending failure modes of BFRC and found that the optimum volume fraction of BF was less than 0.5%. Chen et al. [35] found that the enhancement effect of BF on the splitting tensile strength of BFRC was better when the content of BF was less than 1%. In addition, some scholars believe that BF can also improve the durability of concrete [36,37]. Algin et al. [38] conducted accelerated penetration tests and found that BF reduced the porosity and permeability of the specimen and improved the compactness of BFRC. Niu et al. [39] found that a suitable amount of BF can enhance the chloride resistance of specimens, but excessive addition of BF can have adverse effects.

Cast-in-situ concrete structures with good integrity, flexible size selection, and convenient-to-use local materials are widely used in roads, tunnels, and pile foundations. However, cast-in-situ concrete structures are in contact with the surrounding environment during the pouring process, making them more susceptible to deterioration from the corrosive environment [40,41,42]. Especially for cast-in-situ concrete structures used in underground infrastructure facilities located in salt lakes and saline soil areas, the structures are exposed to corrosive ions from the beginning of service. Corrosive ions intrude into the interior of the structure through diffusion, infiltration, and adsorption, causing potential external sulfate attack (ESA). In addition, some remote saline soils or coastal areas are very scarce in freshwater resources and aggregates and, thus, have to use water or aggregates containing corrosive ions to cast concrete. Such a process would result in concrete corrosion occurring simultaneously with the cement hydration process. Moreover, cast-in-situ concrete structures in this region are also susceptible to the intrusion of corrosive ions from the environment during construction, for example, during the casting process of underground structures such as grouted piles. If protective measures are not taken, the disturbance of the environment during the casting process will cause highly saline soils to fall from the borehole wall, resulting in corrosive ions from the environment entering the interior of the structure and bringing potential internal chloride attack (ICA) [43].

The deterioration mechanism of cement under sulfate and chloride corrosion has been relatively well defined [44,45,46,47,48]. The mechanism of sulfate corrosion is SO_4_^2−^, which reacts with aluminum phases in cement paste to form corrosion products. This process may cause expansion and cracking and, ultimately, lead to damage to structures [49]. The corrosive effect of chloride on concrete is mainly to cause corrosion of steel reinforcement. Furthermore, the corrosive reaction between chloride and cement paste would also cause the spalling of concrete cover [50]. Nevertheless, due to the interactive relationship between sulfate and chloride, concrete deterioration under combined attack is different from that under a single damage factor. Wang et al. [51] found that steel reinforcement corrosion was faster when the specimen was exposed only to chloride. Sulfate reduced the amount of free chloride ions that diffused toward the steel bar region. Sotiriadis et al. [52] found that chloride would reduce the corrosion degree by sulfate and inhibit the corrosion deterioration of concrete. However, Zhang et al. [53] found that sulfate corrosion would decrease the binding capacity of chloride ions by 18%. Abdalkader et al. [54] found that a sulfate attack would reduce the corrosion resistance of steel reinforcement and exacerbate the corrosion deterioration of the specimen. However, there are few studies on the deterioration mechanism of cast-in-situ concrete under combined impact. Therefore, it is necessary to conduct research in this area.

BF is a material with very broad application prospects. Many researchers have studied its mechanical properties and durability. Niu et al. [39] found that the chloride diffusion coefficient of concrete containing 0.05% BF and 0.1% polypropylene fiber decreased by 77.8%. When the fiber volume fraction exceeds 0.15%, the chloride ion diffusion coefficient gradually increases. Xu et al. [55] chose BF-reinforced concrete and ordinary concrete and studied their mechanical properties under three kinds of freeze–thaw conditions (water, 3% NaCl, and 5% Na_2_SO_4_). It was found that when the BF content was 0.15%, the frost resistance was the best. Wang et al. [56] studied the performance of BF-reinforced concrete under the erosion of magnesium sulfate and sodium sulfate and found that excessive fiber would have an adverse effect on durability. When the BF parameter is about 0.1%, it is the most economical and reasonable. Fan et al. [26] studied the effect of BF on the durability of concrete with internal magnesium and external sulfate attack through experiments. The results showed that the flexural strength of 0.5% BF specimens increased by 16.2% when exposed to external sulfate–internal magnesium composite attack for a long time. At present, there is no clear conclusion on whether BF can improve the performance of concrete under the combined attack of chloride salt and sulfate. Therefore, it is very meaningful to carry out research in this field.

Researchers have conducted outstanding and numerous works on the corrosion deterioration mechanism of concrete and the mechanical properties of BFRC and have made admirable periodic progress. However, existing research mostly focuses on prefabricated concrete or mortar components, while research on corrosion deterioration of basalt fiber-reinforced cast-in-situ concrete under sulfate, chloride, and combined attack is relatively rare. The deterioration mechanism of basalt fiber-reinforced cast-in-situ concrete is still uncertain. In this study, concrete specimens were prepared and soaked in different solutions for 90 days. Internal attack is simulated by premixing chloride in specimens, and external attack is simulated by placing specimens in different corrosive environments. Combined with the above literature survey results, it is found that when the BF content is less than 0.5%, the durability of concrete is the best. Therefore, different contents of BF (varying from 0.1% to 0.5%) were premixed in concrete specimens to study their effect on improving corrosion resistance. The appearance, size, mass, flexural, and compressive strength of specimens were investigated during the soaked period to determine the changes in the physical and mechanical properties of specimens. Moreover, SEM, EDS, XRD, and TG/DTG analyses were conducted to observe the microstructural and mineralogical changes in specimens.

## 2. Materials and Methods

### 2.1. Materials

**Figure 1:**
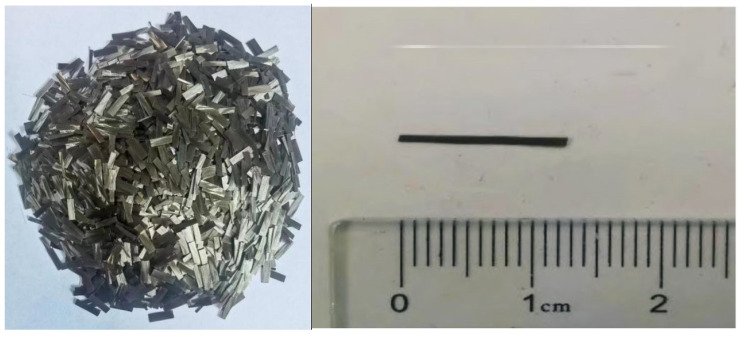
Basalt fiber used in the present study.

In this study, Portland cement (P.O. 42.5, made in China) was used to prepare concrete specimens. Table 1 lists the chemical composition of cement. Pebble with a diameter of 5–10 mm and river sand with a fineness modulus of 2.6 mm were used as coarse aggregate and fine aggregate, respectively. The mixing water used for preparation of the specimens, as well as the soaking solution, was distilled water. AR Na_2_SO_4_ and NaCl (produced by Shanghai Sinopharm) were used to simulate internal corrosion and to prepare solutions. Basalt fiber (produced by Changsha, Hunan Province, China) was used to mix in concrete and the photo is shown in Figure 1. Table 2 lists the physical properties of basalt fiber. Table 3 lists the mixture proportion of concrete specimens.

### 2.2. Specimen and Solution Preparation

To ensure that BF was evenly distributed in specimens, coarse aggregate and fine aggregate were weighed and dry-mixed for 30 s at first. Then, we poured the weighed BF evenly into the mixer and mixed for 60 s. Finally, we added cement and distilled water and mixed for 120 s. We poured freshly mixed concrete into different molds and fully vibrated them on a vibrating table. To simulate the corrosion process of cast-in-situ concrete, the specimens, along with the molds, were soaked in the soaking solution after 2 h of standard curing. After 24 h, we removed the molds and soaked the specimens in the corrosion solutions again. The temperature during the immersion period was 20 ± 2 °C. In this study, 40 × 40 × 160 mm prismatic and 100 × 100 × 100 mm cubic concrete specimens were prepared for flexural test and compressive test, respectively, in which the prismatic specimen is used for flexural test and the cubic specimen is used for compressive test. To simulate the corrosion process of cast-in-situ concrete structures in salt lakes and saline soil areas, internal corrosion was achieved by adding 3% (mass fraction) chloride in the specimen. In this paper, two immersion environments were set up: 10% (mass fraction) Na_2_SO_4_ solution and distilled water. To ensure that the specimens are completely soaked in the solution, the liquid level of the immersion pool should be 30 mm above the specimen. Checks and adjustment of the immersion pool solution concentration were performed weekly. Details of specimens and slump are presented in Table 4 [57].

### 2.3. Test Methods

#### 2.3.1. Physical Properties

Photographs of the appearance of specimens were recorded using a digital camera. The size of specimens was determined by a dial indicator (produced by Hitachi, Tokyo, Japan) with an accuracy of 0.05 mm, using a copper limiter embedded in 40 × 40 × 160 mm specimens. The mass of specimens was determined by an electronic balance (produced by Meilen, Shenzhen, China) with an accuracy of 0.001 g. The size and mass were obtained by measuring 3 times and recording the average value. Afterward, the size and mass change ratios were calculated. Change ratios are calculated by (X_t_ − X_0_)/X_0_ (in which X_t_ is the measured value at different times and X_0_ is the initial value of size or mass).

#### 2.3.2. Mechanical Properties

The flexural strength of specimens was determined by the YZs-300 bending testing machine (produced by Jianyi Instrument Machinery Co., Ltd., Wuxi, China), and the loading rate was set to 0.05 MPa/s during the test. The compressive strength of specimens was determined by the TYE-3000 compression testing machine (produced by Jianyi Instrument Machinery Co., Ltd., Wuxi, China), and the loading rate was set to 0.5 MPa/s during the test. In addition, the surface of specimen in direct contact with test machine was carefully polished with abrasive paper.

#### 2.3.3. Microstructural and Mineral Properties

**Figure 2:**
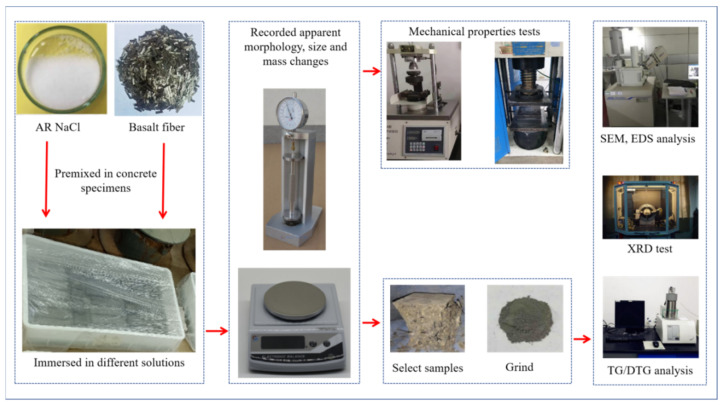
Test flowchart.

Specimens were taken out at different corrosion times and put into isopropyl alcohol to terminate the reaction. Then, we put the specimens in an oven at 60 °C (low-temperature drying to prevent the decomposition of corrosion products) for 48 h. Microstructural change in specimen was observed using ZEISS-SIGMA 300 system (produced by ZEISS Sigma, Oberkochen, Germany). EDS analysis was also performed to further determine the elemental composition of observed product. Mineral analysis of specimens was conducted by XRD analysis using Model RINT 2000 X-ray diffractometer (produced by Rigaku Tokyo, Japan) and TG/DTG analysis using Model NETZSCH-STA 449C system (produced by NETZSCH, Bavaria, Germany). The samples used for SEM analysis were core samples 5 mm away from the corrosion surface. The samples used to conduct XRD and TG/DTG analyses were milled core samples powder. XRD analysis was performed with the following testing parameters: 40 kV, 100 mA, Cu target, 5 °/min scanning speed from 5 to 65 (2θ). TG/DTG analysis was performed with the following testing parameters: heating rate 20 °C/min, temperature ranges from 30 °C to 1000 °C, nitrogen atmosphere. The flow chart of the test is depicted in Figure 2.

## 3. Results and Discussion

### 3.1. Microstructural and Mineral Properties

#### 3.1.1. Mineral Properties

**Figure 3:**
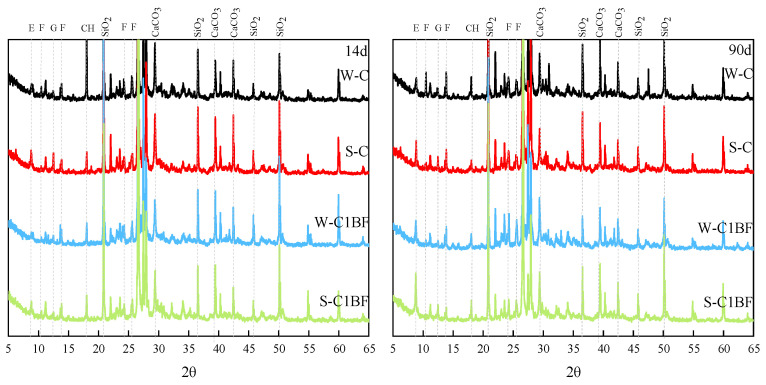
XRD analysis results of specimens with different corrosion times.

**Figure 4:**
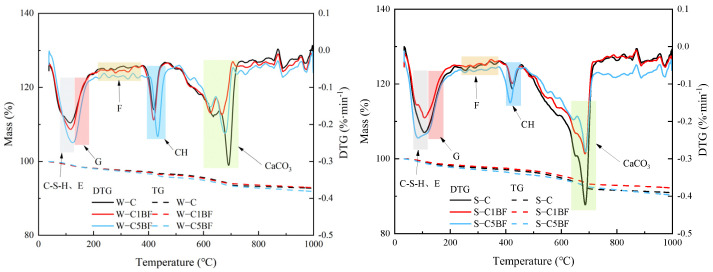
TG/DTG analysis results of specimens after soaking for 90 days. (Note: E represents ettringite, G represents gypsum, F represents Friedel’s salt, and CH represents calcium hydroxide).

XRD and TG/DTG analyses were performed to determine the changes in the mineral composition of the specimen during immersion; the analysis results are shown in Figure 3 and Figure 4. The E, G, CH, and F in Figure 3 and Figure 4 stand for ettringite, gypsum, Ca(OH)_2_, and Friedel’s salt, respectively. From Figure 3, diffraction peaks of gypsum and ettringite are observed in specimens. In addition, diffraction peaks of chloride corrosion products are also obvious as the specimens were premixed with chloride. With increasing corrosion time, an increasing amount of ettringite and Friedel’s salt are observed, indicated by their diffraction peaks. Moreover, the diffraction peaks of gypsum and ettringite are higher for specimens soaked in Na_2_SO_4_ solution, indicating that more products are generated in the specimens under external sulfate attack.

From Figure 4, the peaks in the range of 90–120 °C are related to the decomposition of ettringite and C-S-H gel. And the peaks produced by gypsum decomposition are mainly in the range of 130–150 °C. Furthermore, the peaks produced by Friedel’s salt decomposition are found in the range of 280–380 °C [58]. The peaks in the range of 400–500 °C and 650–750 °C are attributed to the decomposition of Ca(OH)_2_ and CaCO_3_, respectively.

The diffraction peaks of Friedel’s salt in Figure 3 require attention. The diffraction peaks of the specimen soaked in Na_2_SO_4_ solution are lower than the specimen soaked in distilled water, indicating that sulfate competes with chloride and limits the formation of chloride-induced products, especially under the long-term corrosion of sulfate. Some scholars have found that sulfate attack can lead to the decomposition of Friedel’s salt and the stability of Friedel’s salt is easily affected by sulfate attack [59]. Similar results can also be obtained in the TG/DTG analysis. As illustrated in Figure 4, the peaks of gypsum and ettringite show very similar results regardless of which solution the specimen is soaked in. It is shown that chloride and sulfate have a competitive relationship and restrict each other in inducing the formation of corrosion products.

#### 3.1.2. Microstructures

**Figure 5:**
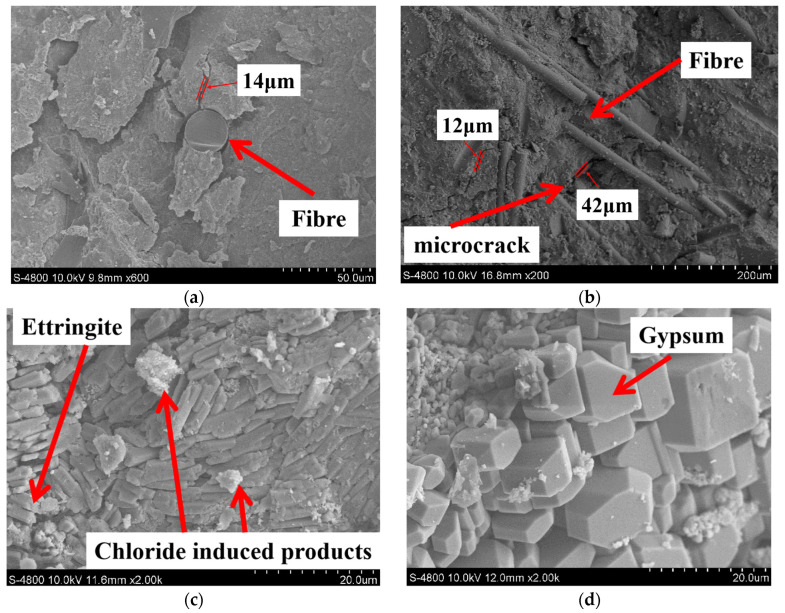
SEM images of specimens, (**a**) W-C, (**b**) W-C1BF, (**c**) W-C3BF, (**d**) W-C5BF.

**Figure 6:**
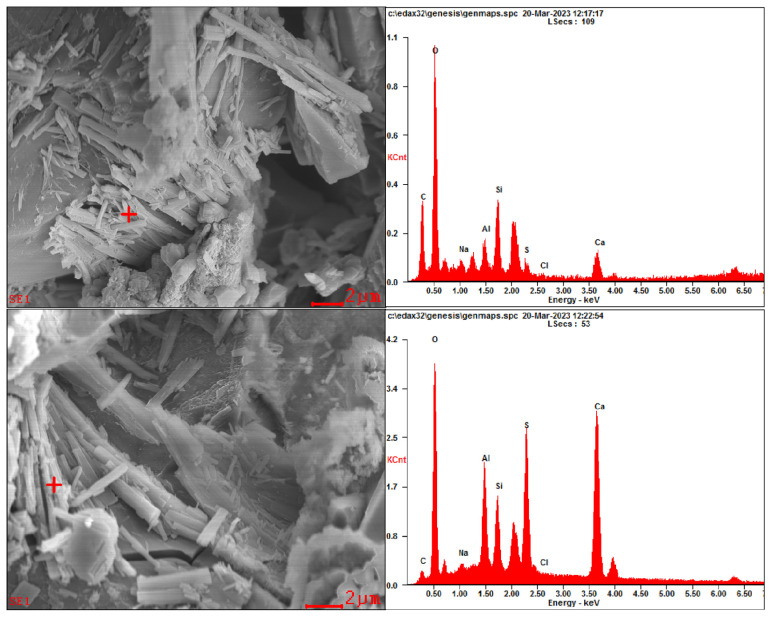
EDS analysis results on SEM images for specimens.

Representative SEM images are presented in Figure 5. In addition, EDS analysis was also performed to determine the elemental composition of specific crystals; the results are presented in Figure 6.

From Figure 5 and Figure 6, under the corrosion of aggressive ions from both internal and external sources, corrosion products are found in the pores and cracks of specimens, such as gypsum and ettringite. In addition, according to the EDS analysis results, a relatively high peak value of elemental chlorine in the corrosion products can be found, demonstrating that chlorides participate in the generation of corrosion products in the specimen.

From Figure 5a, it can be seen that BF is wrapped with concrete, and the combination of the fiber–cement matrix interface is relatively tight. BF fills some of the initial pores and improves the compactness of the cement paste. In addition, from Figure 5b, microcracks are distributed within the concrete, and a large amount of BF is distributed around the microcracks and forms a network structure. The BF at both ends of the microcracks inhibits the development of microcracks, preventing microcracks connection from forming macroscopic cracks. Moreover, the presence of BF can also limit cracking and block the passage of external corrosion solution into concrete [60]. The early shrinkage of concrete leads to internal defects, providing channels for the diffusion and invasion of corrosive ions. The premixed BF would improve the pore structure and water loss rate of concrete. This can reduce early shrinkage and improve the durability of concrete structures [61].

From Figure 5c,d, corrosion products induced by sulfate and chloride are observed in SEM images. Sulfate and chloride corrosion occur simultaneously when sulfate and chloride jointly invade the concrete. The sulfate attack of concrete is a very complicated physicochemical process. Its main mechanism is that SO_4_^2−^ in the environment invades the cement-based material matrix and reacts with hydration products, such as Ca(OH)_2_ and hydrated calcium aluminate, to generate gypsum and ettringite (as illustrated by Equations (1)–(4)) [62,63,64]. This process would cause an increase in the solid phase volume of concrete and generate large expansion pressure inside the concrete, resulting in concrete cracks and failure of structures.

(1) $${\mathrm{Ca}(\mathrm{OH})}_{2}+{\mathrm{Na}}_{2}{\mathrm{SO}}_{4}+2H_{2}O\to {\mathrm{CaSO}}_{4}\cdot 2H_{2}O+2\mathrm{NaOH}$$

(2) $$3{\mathrm{Na}}_{2}SO_{4}+3\mathrm{CaO}\cdot {\mathrm{Al}}_{2}O_{3}+3{\mathrm{Ca}(\mathrm{OH})}_{2}+32H_{2}O\to 3\mathrm{CaO}\cdot {\mathrm{Al}}_{2}O_{3}\cdot 3\mathrm{CaS}O_{4}\cdot 32H_{2}O+6\mathrm{NaOH}$$

(3) $$3\mathrm{CaO}\cdot {\mathrm{Al}}_{2}O_{3}+3\left({\mathrm{CaSO}}_{4}\cdot 2H_{2}O\right)+26H_{2}O\to 3\mathrm{CaO}\cdot {\mathrm{Al}}_{2}O_{3}\cdot 3\mathrm{CaS}O_{4}\cdot 32H_{2}O$$

(4) $$4\mathrm{CaO}\cdot {\mathrm{Al}}_{2}O_{3}\cdot 13H_{2}O+3\left({\mathrm{CaSO}}_{4}\cdot 2H_{2}O\right)+14H_{2}O\to 3\mathrm{CaO}\cdot {\mathrm{Al}}_{2}O_{3}\cdot 3\mathrm{CaS}O_{4}\cdot 32H_{2}O+\mathrm{CaO}\cdot H_{2}O$$

For chloride-induced attack, corrosion of steel reinforcement in concrete structures exposed to marine or deicing salt environments is a recognized durability problem. In addition, chloride can also react with cement hydration products to generate corrosion products and cause alkali-aggregate reactions, greatly reducing the durability of concrete [65]. The chloride corrosion products have poor physico-mechanical properties that can negatively affect concrete or mortar. Its main mechanism is that Cl^−^ reacts with calcium compounds in the concrete to form products such as hydrated calcium silicate (as illustrated by Equation (5)), causing the concrete to spall [66].

(5) $$3\mathrm{CaO}\cdot {\mathrm{Al}}_{2}O_{3}\cdot {\mathrm{CaSO}}_{4}\cdot 12H_{2}O+2\mathrm{NaCl}\to 3\mathrm{CaO}\cdot {\mathrm{Al}}_{2}O_{3}\cdot \mathrm{CaC}l_{4}\cdot 10H_{2}O+{\mathrm{Na}}_{2}{\mathrm{SO}}_{4}+2H_{2}O$$

### 3.2. Physical Properties

#### 3.2.1. Appearance Change

**Figure 7:**
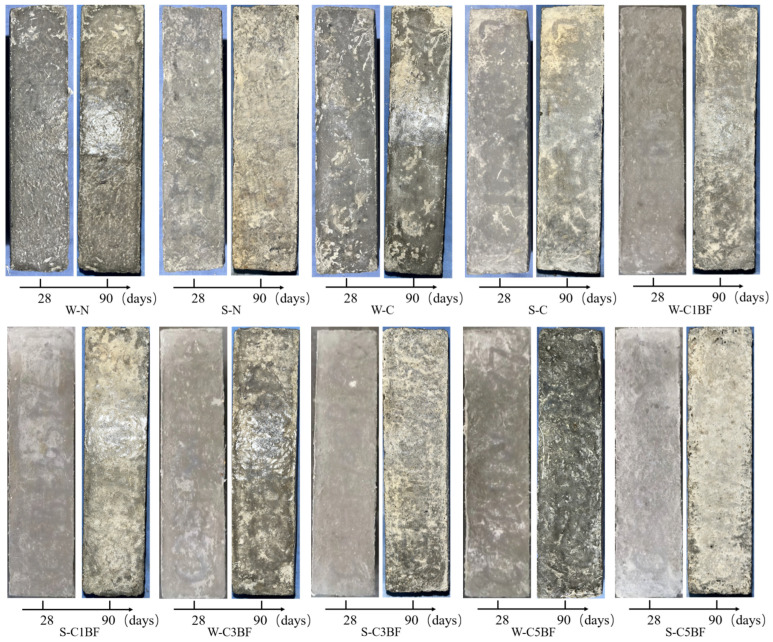
Appearance of specimens with different corrosion times.

The photographs of specimens with different corrosion times are presented in Figure 7. It is obvious that there is a significant difference in the degree of deterioration of the specimens suffering different corrosion conditions. It can be observed that corrosion degradation is more severe for specimens immersed in Na_2_SO_4_ solution; spalling of concrete powder on the specimen surface is more obvious. In addition, the surface of specimens premixed with chloride but soaked in distilled water also shows partial concrete powder spalling under the internal chloride attack.

It is remarkable that the most severe surface peeling is observed on the specimens premixed with chloride and soaked in Na_2_SO_4_ solution, both suffering ESA-ICA combined attack. The spalling of concrete powder on the specimen surface can be observed by the naked eye. Large areas of concrete powder spalling on the specimen surface result in an unusual white color on the specimen surface, indicating that premixed chloride exacerbates the deterioration of concrete induced by external sulfate attack.

#### 3.2.2. Size and Mass Changes

**Figure 8:**
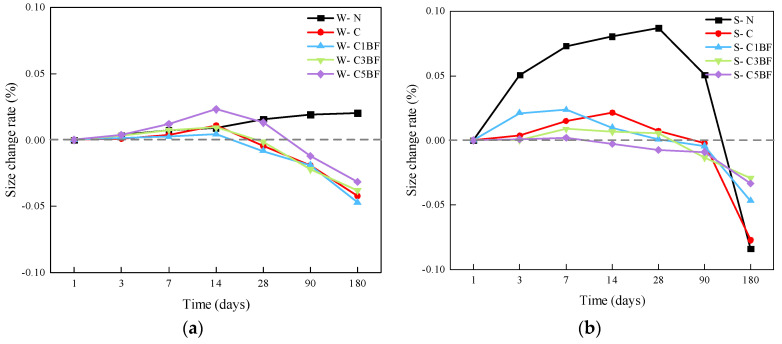
Size changes in specimens over immersion time, (**a**) Soak the specimen in water, (**b**) Soak the specimen in a corrosive solution.

The size change rates of the specimens soaked in distilled water and Na_2_SO_4_ solution are presented in Figure 8a and Figure 8b, respectively.

From Figure 8a, it can be seen that the size of the W-N group increases gradually. In addition, the size of the specimens premixed with chloride increases in the first 14 days and then decreases. And except for the W-C5BF group, the size of all other specimens premixed with chloride is lower than the initial value at 28 days. The possible reason for this is that chloride corrosion products first fill the initial pores within concrete. As the corrosion reaction progresses, the accumulation of corrosion products results in insufficient space within the pores to accommodate the newly generated corrosion products, causing the detachment of the surface layer of concrete, which results in a reduction in the growth rate of the specimen size.

From Figure 8b, the size of the S-N group increases rapidly during the first 28 days and then decreases rapidly. This is due to the large amount of ettringite generated inside the specimen under the external sulfate attack. It is well-accepted that the generation of ettringite inside concrete would result in rapid expansion at an early stage. However, the long-term external sulfate attack would cause the concrete spalling, resulting in a decline in specimen size. For specimens premixed with chloride, the size changes in specimens are smoother, indicating that the premixed chloride would affect the corrosion effect of sulfate on concrete. C_3_A in cement paste would preferentially react with chloride when chloride and sulfate coexist in the solution. The chloride corrosion products would fill the pores within concrete, block the corrosion channel for sulfate, and greatly slow down the sulfate intrusion speed into concrete [52]. In addition, it can be seen that compared to the S-C group. S-C1BF, S-C3BF, and S-C5BF groups show lower size growth at an early age and lower size reduction after 180 days. The size of the specimens is more “stable” during the corrosion period. Furthermore, increasing the amount of BF makes this phenomenon more significant. As stated earlier, BF would prevent the generation and expansion of microcracks in concrete. Heterogeneously distributed fibers not only inhibit the early shrinkage deformation of concrete but also disperse the energy generated during the hardening process of concrete. Therefore, BF can sufficiently reduce the stress concentration at the tip of microcracks inside the concrete and prevent the expansion of microcracks [60,61], thereby reducing the width, length, and area of concrete cracks and delaying the penetration of sulfate from the external environment to internal concrete.

**Figure 9:**
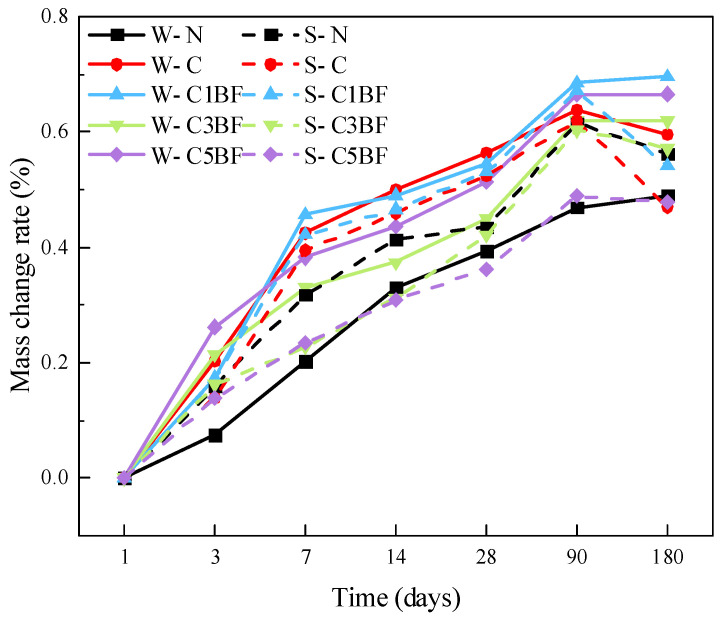
Mass changes of specimens over immersion time.

Mass change ratios are presented in Figure 9. Mass change is a useful parameter to reflect the surface damage of concrete under various salt corrosion. It can be seen that the mass of specimens soaked in distilled water increases continuously, except for the W-C group. This is due to the flaking of the specimen surface under chloride corrosion, which results in a reduction of the specimen mass. The mass of specimens soaked in Na_2_SO_4_ solution increases in the first 90 days and decreases at 180 days. The S-C group suffers the most severe mass loss under the coupling corrosion of sulfate and chloride, with a 15% reduction in mass at 180 days compared to 90 days. Furthermore, it can be concluded that the degree of mass loss is mitigated by internal fiber blending, and the effect is more effective with higher fiber content.

It is generally believed that corrosive ions from external corrosion solutions would invade the pores of concrete. Corrosion products generated by the reaction of corrosive ions with cement would result in an increase in specimen mass [67]. However, it should be noted that for specimens with the same internal corrosion conditions, the mass growth rate of specimens soaked in distilled water is higher than that of specimens soaked in Na_2_SO_4_ solution, except for the W-N and S-N groups. The reason for this phenomenon could be that the specimens premixed with chloride and soaked in Na_2_SO_4_ solution have already spelled on the surface during the corrosion process (as shown in Section 3.2.1). Spalling of concrete powder from the specimen surface results in a reduction in the growth rate of specimen mass.

### 3.3. Mechanical Properties

#### 3.3.1. Flexural Strength

**Figure 10:**
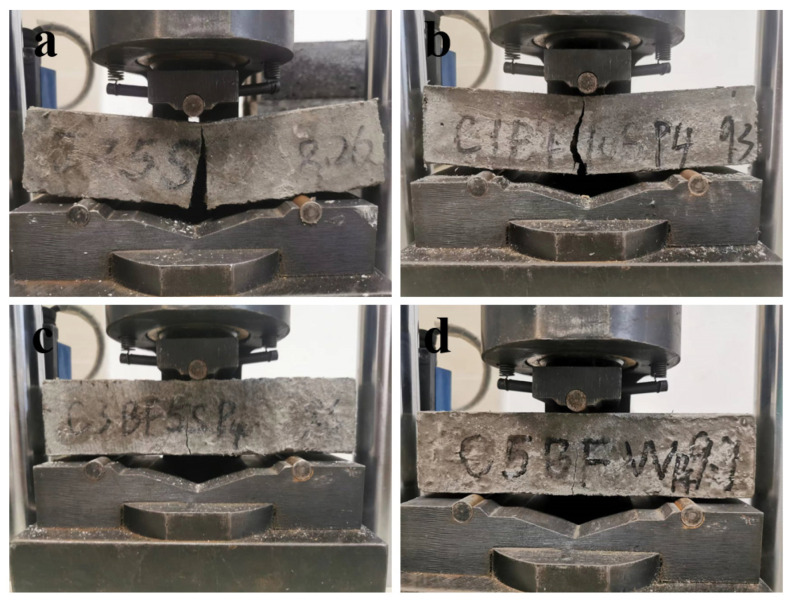
Flexural failure photos of samples with different BF content: (**a**) W-C, (**b**) W-C1BF, (**c**) W-C3BF, (**d**) W-C5BF.

**Figure 11:**
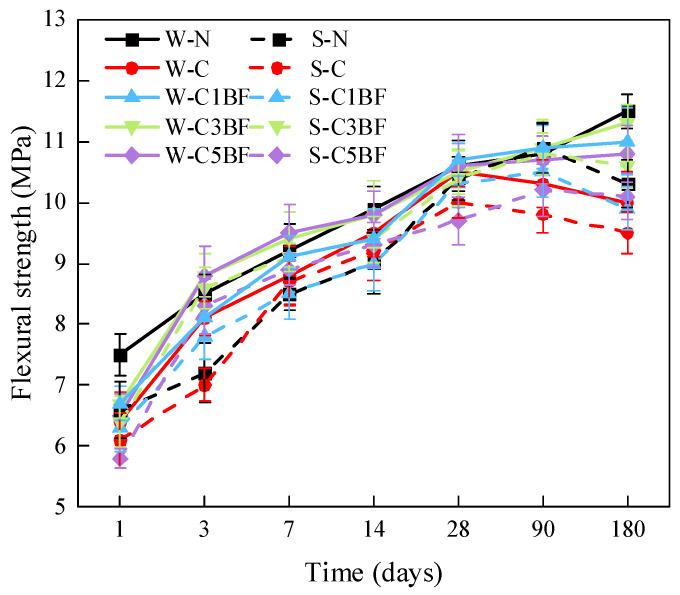
Flexural strength changes of specimens over immersion time.

Figure 10 shows the bending failure photos of specimens with different BF contents. The flexural strength of specimens with different corrosion times is shown in Figure 11. According to Figure 10, it can be found that with the increase in BF content, the brittle failure of the sample gradually slowed down, and the integrity of the sample gradually increased. From Figure 11, the flexural strength of specimens soaked in distilled water is higher than that of specimens immersed in Na_2_SO_4_ solution when the internal corrosion conditions are the same, demonstrating that the specimens immersed in Na_2_SO_4_ solution are degraded more seriously than those soaked in distilled water. In addition, the development trend of flexural strength with time is significantly different for different specimens. According to the trend of evolution of flexural strength, the specimens can be divided into three groups. (a) W-N, W-C1BF, W-C3BF, and W-C5BF group, the flexural strength of specimens increases continuously with time. (b) S-N, S-C1BF, S-C3BF, and S-C5BF group, the flexural strength of specimens increases over time within 90 days and decreases at 180 days. (c) W-C and S-C group, the flexural strength of specimens continues to increase with time for 28 days and then decreases.

From group (c), it can be observed that premixed chloride has an adverse influence on the development of flexural strength of specimens, causing a decrease in flexural strength after 28 days. Furthermore, an external sulfate attack would further exacerbate the adverse influence of chloride on the development of the flexural strength of specimens. Moreover, from groups (a) and (b), it can be concluded that the premixed BF can alleviate the adverse influence of chloride on the flexural strength, making the development trend of flexural strength of specimens close to that of the N (W-N and S-N) group. In addition, increasing the fiber content has beneficial influences, especially in the 14 days. The flexural strength of the C3 (W-C3BF and S-C3BF) and C5 (W-C5BF and S-C5BF) groups is close to or even higher than the flexural strength of the N (W-N and S-N) group. On the whole, the S-C5BF group has the best reinforcement effect, and the 180-day flexural strength is 11.6% higher than that of the S-C group.

#### 3.3.2. Compressive Strength

**Figure 12:**
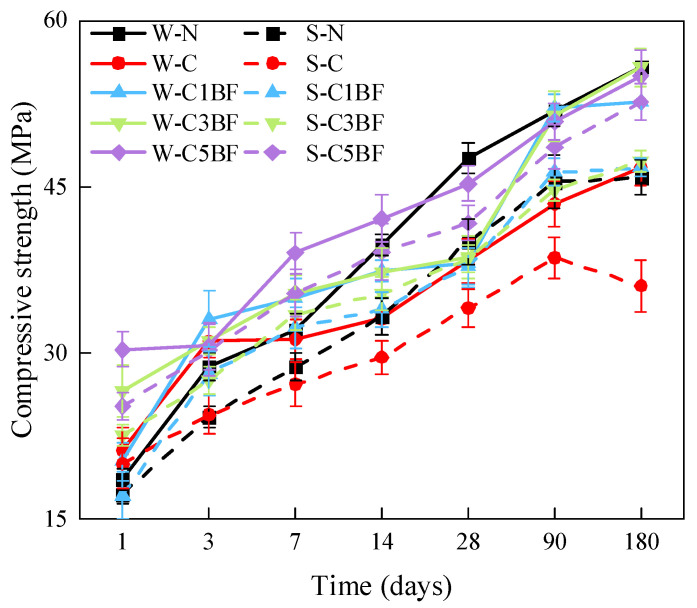
Compressive strength changes in specimens over immersion time.

The compressive strength of specimens with different corrosion times is shown in Figure 12. Generally, the compressive strength of specimens with the same internal corrosion conditions soaked in distilled water is higher than those soaked in Na_2_SO_4_ solution. Furthermore, the compressive strength of specimens all increase with time except for the S-C group. From the comparison of the W-N group with the S-N group as well as with the W-C group, it can be concluded that both external sulfate attack and internal chloride attack have a detrimental influence on the compressive strength development of specimens. From Figure 12, it can be seen that the W-C group and S-C group have lower compressive strength in the early stage, and the strength at 28 days is 15.4% and 24.8% lower than that of the W-N group, respectively. The S-C group suffers the most severe corrosion deterioration under the ESA-ICA combined attack, with a 6% reduction in compressive strength at 180 days compared to 90 days. Moreover, similar to flexural strength, the premixed BF mitigates the detrimental influence of chloride on the compressive strength, making the compressive strength of specimens premixed with chloride close to or even higher than that of the N (W-N and S-N) group. Additionally, the increase in fiber content has a positive influence on the development of compressive strength of specimens.

By comparing the results of flexural and compressive strength, both external sulfate attack and internal chloride attack have a detrimental influence on the strength development of the specimen. The specimen subjects the most severe corrosion deterioration under the ESA-ICA combined attack, with a significant decrease in mechanical properties. Either external sulfate erosion or internal chloride erosion would cause the spalling of concrete powder on the specimen surface (as shown in Section 3.2.1). This is very detrimental to the strength development of concrete structures. Furthermore, penetrated corrosive ions would react with cement hydration products immediately. Sulfate- and chloride-induced corrosion products would accumulate in the initial pores and weak joint areas of concrete. The continuous accumulation of corrosion products in the pores would lead to pore expansion and destruction, which would cause the generation of internal microcracks. The sulfate penetration in pores would also cause the formation of macroscopic cracks within the concrete, which would negatively influence the physical and mechanical properties of specimens. According to the fiber spacing theory, the destruction process of concrete occurs first at the bond between mortar and stone or at the interfacial cracks that formed during the hardening process of cement. Premixed BF would prevent the generation and expansion of microcracks, delay the intrusion of corrosive ions, and enhance the durability of concrete in corrosive environments. Furthermore, incorporating a certain amount of BF into concrete can form a network structure in the concrete interface layer [68]. This network structure can transfer part of the stress to fibers when concrete is subjected to external forces and has a role in bridging cracks to enhance the mechanical properties of concrete. As shown in Section 3.1.2, BF is dispersed during the preparation of the concrete and forms a network structure with a certain support capacity inside the concrete. The network structure can bridge damaged areas and, thus, reduce crack propagation rate, thus increasing the strength of concrete [69].

### 3.4. Sulfate Concentration

**Figure 13:**
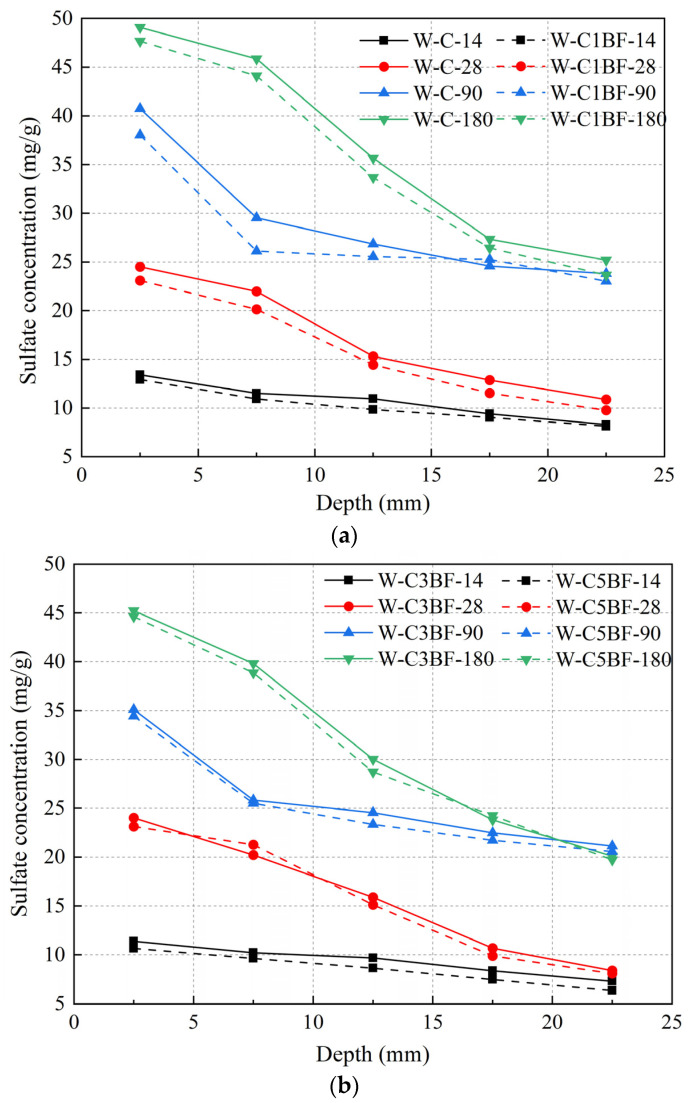
Sulfate concentrations of specimens at different immersion times: (**a**) W-C and W-C1BF and (**b**) W-C3BF and W-C5BF.

The sulfate concentration of different samples at different depths was determined by chemical titration, and the results are shown in Figure 13. The powder is drilled every 5 mm, and the abscissa in Figure 13 is the average value of the drilling. The early hydration of cast-in-situ concrete is incomplete, and the existence of original defects and pores leads to the rapid diffusion of sulfate. As shown in Figure 13, the sulfate concentration decreased with the increase in the depth under the exposed surface of the sample. The concentration of 0–5 mm increased significantly at 180 days, while the concentration of 20–25 mm was similar to the data of 90 days. In general, changes in appearance can affect the diffusion of sulfate. Cracks in concrete increase the diffusion path of corrosive ions and accelerate the degradation of concrete. The degree of degradation of the sample pre-mixed with Cl^−^ is more serious, as shown in Figure 7, which correspondingly accelerates the diffusion rate of sulfate, resulting in an increase in sulfate concentration. As mentioned above, the addition of BF limits the development of the crack system and alleviates the corrosion channel of the external sulfate to a certain extent. Therefore, the sulfate concentration of BF-premixed concrete is lower than that of W-C.

## 4. Conclusions

Concrete specimens with or without premixed chloride were prepared and soaked in different solutions to study the corrosion deterioration mechanism of cast-in-situ concrete structures in different corrosive environments. Furthermore, the effect of basalt fiber in corrosive environments to enhance the corrosion resistance of cast-in-situ concrete was also studied. Physical, mechanical, mineral, and microstructure properties of the specimens during the soaked period were measured. The deterioration mechanism and the protective effect of basalt fiber were also discussed and revealed. According to the results and discussion in this paper, the main conclusions are as follows:

1. Premixed chloride has a significant detrimental influence on the strength development of cast-in-situ concrete. Concrete powder spalling occurs on the surface of the specimen under the ICA.
2. Although chloride is in competition with sulfate, the most severe corrosion of the specimens occurs under the ESA-ICA combined attack. Large areas of concrete powder spalling occur on the surface of the specimen, and the strength development of the specimen is severely affected.
3. Increasing fiber content is beneficial for enhancing its effectiveness when the fiber content is less than 0.5%. After premixing with BF, the physical and mechanical properties of cast-in-situ concrete are improved. BF mainly plays the role of reinforcement and improvement. In terms of strengthening, BF filled the original defects of the specimen and improved the bearing capacity of the strength specimen. In terms of crack resistance, it limits the development of cracks and reduces stress concentration at the crack tip.
4. The filling and bridging effects of basalt fibers alleviate the negative effects of internal and external corrosion. When the basalt fiber content is 0.5%, the flexural strength of the specimen is increased by 16.2%. Results show that BF can reduce the internal pores of concrete and improve the corrosion resistance of concrete. Therefore, it is feasible to premix BF into cast-in-situ concrete to improve the corrosion resistance and mechanical properties of concrete.

## Figures and Tables

**Table 1:** Chemical compositions of cement.

Chemical Composition	Al_2_O_3_	SiO_2_	SO_3_	Cl	TiO_2_	Fe_2_O_3_	Na_2_O	K_2_O	MgO	CaO
Content (%)	5.64	18.48	2.40	0.08	0.51	3.63	0.31	0.68	0.75	67.21

**Table 2:** Physical properties of basalt fiber.

Material	Length (mm)	Diameter (μm)	Tensile Strength (MPa)	Elastic Modulus (GPa)	Density (g/cm^3^)
Basalt fiber	12	17	3000–4800	90–100	2.80

**Table 3:** Mixture proportion of concrete.

W/C	Cement (kg/m^3^)	Water (kg/m^3^)	Sand (kg/m^3^)	Pebble (kg/m^3^)
0.46	478	220	629	983

**Table 4:** Details information of specimens.

Specimens in Distilled Water	Specimens Soaked in Na_2_SO_4_ Solution	Mixed Salt	Mixed BF	Number of Samples	Slump (cm)
W-N	S-N	No	No	42	6.8
W-C	S-C	3% Cl^−^	No	42	8.2
W-C1BF	S-C1BF	3% Cl^−^	0.1%BF	42	7.4
W-C3BF	S-C3BF	3% Cl^−^	0.3%BF	42	4.6
W-C5BF	S-C5BF	3% Cl^−^	0.5%BF	42	2.2

## Data Availability

Data will be made available upon request.
